# Pathogenesis of chronic rhinosinusitis with nasal polyps: role of IL-6 in airway epithelial cell dysfunction

**DOI:** 10.1186/s12967-020-02309-9

**Published:** 2020-03-24

**Authors:** Emilie Bequignon, David Mangin, Justine Bécaud, Jennifer Pasquier, Christelle Angely, Mathieu Bottier, Estelle Escudier, Daniel Isabey, Marcel Filoche, Bruno Louis, Jean-François Papon, André Coste

**Affiliations:** 1 Service d’Oto-Rhino-Laryngologie et de Chirurgie cervico-faciale, AP-HP, Hôpital Henri Mondor et Centre Hospitalier Intercommunal de Créteil, 94010 Créteil, France; 2 INSERM, U955, Equipe 13, Faculte de Medecine, 8 rue du General Sarrail, 94010 Créteil, France; 3 Faculté de Médecine, Université Paris-Est, 94010 Créteil, France; 4 CNRS ERL 7000, 94010 Créteil, France; 5 Nice Breast Institute, 06000 Nice, France; 6 Stem Cell & Microenvironment Laboratory, Weill Cornell Medicine-Qatar, Doha, Qatar; 7 Inserm U933, Paris, France; 8 Université Pierre et Marie Curie, Paris, France; 9 Service de génétique et d’embryologie médicale, AP-HP Hôpital Armand-Trousseau, Paris, France; 10 Service d’Oto-Rhino-Laryngologie et de Chirurgie cervico-faciale, AP-HP, Hôpital Bicêtre, 94270 Le Kremlin-Bicêtre, France; 11 Faculté de Médecine, Université Paris-Sud, 94275 Le Kremlin-Bicêtre, France

**Keywords:** Nasal Polyps, IL-6, Interleukin 6, Inflammation, Repair mechanisms, Mucociliary clearance, Chronic rhinosinusitis with nasal polyps (CRSwNP), Wound healing, Epithelial cell, IL-9

## Abstract

**Background:**

Chronic rhinosinusitis with nasal polyps (CRSwNP) is characterized by an alteration in airway epithelial cell functions including barrier function, wound repair mechanisms, mucociliary clearance. The mechanisms leading to epithelial cell dysfunction in nasal polyps (NPs) remain poorly understood. Our hypothesis was that among the inflammatory cytokines involved in NPs, IL-6 could alter epithelial repair mechanisms and mucociliary clearance. The aim of this study was to evaluate the in vitro effects of IL-6 on epithelial repair mechanisms in a wound repair model and on ciliary beating in primary cultures of Human Nasal Epithelial Cells (HNEC).

**Methods:**

Primary cultures of HNEC taken from 38 patients during surgical procedures for CRSwNP were used in an in vitro model of wound healing. Effects of increasing concentrations of IL-6 (1 ng/mL, 10 ng/mL, and 100 ng/mL) and other ILs (IL-5, IL-9, IL-10) on wound closure kinetics were compared to cultures without IL-modulation. After wound closure, the differentiation process was characterized under basal conditions and after IL supplementation using cytokeratin-14, MUC5AC, and β_IV_ tubulin as immunomarkers of basal, mucus, and ciliated cells, respectively. The ciliated edges of primary cultures were analyzed on IL-6 modulation by digital high-speed video-microscopy to measure: ciliary beating frequency (CBF), ciliary length, relative ciliary density, metachronal wavelength and the ciliary beating efficiency index.

**Results:**

Our results showed that: (i) IL-6 accelerated airway wound repair in vitro, with a dose–response effect whereas no effect was observed after other ILs-stimulation. After 24 h, 79% of wounded wells with IL6-100 were fully repaired, vs 46% in the IL6-10 group, 28% in the IL6-1 group and 15% in the control group; (ii) specific migration analyses of closed wound at late repair stage (Day 12) showed IL-6 had the highest migration compared with other ILs (iii) The study of the IL-6 effect on ciliary function showed that CBF and metachronal wave increased but without significant modifications of ciliary density, length of cilia and efficiency index.

**Conclusion:**

The up-regulated epithelial cell proliferation observed in polyps could be induced by IL-6 in the case of prior epithelial damage. IL-6 could be a major cytokine in NP physiopathology.

## Background

Primary nasal polyposis, also called chronic rhinosinusitis with nasal polyps (CRSwNP), is a common disease that affects 4% of the population. Symptoms relate to growth of polyps within the sinonasal cavities and can include hyposmia, nasal congestion, discharge and facial pain [1].

An understanding of the biological mechanisms involved in CRSwNP may inform treatment and diagnostic decisions, but the epithelial and inflammatory processes leading to nasal polyp (NP) growth are currently poorly defined. CRSwNP is characterized by persistent mucosal inflammation which is a result of inappropriate or excessive immune response to foreign agents [2]. Respiratory epithelium in NPs exhibits morphological changes consistent with epithelial dysfunction, including basal cell proliferation, goblet cell hyperplasia and the loss of differentiation of ciliated cells [3–7]. A recent review [8] describes how dysfunction of the airway epithelium contributes to the pathogenesis of CRSwNP and suggests that mucociliary clearance [9] and maintenance barrier function [10] could be altered in NP epithelial cells. Furthermore, dysregulation of epithelial wound repair with an inability to obtain *ad integrum* repair of the nasal airway epithelium has been described in NPs [3, 11, 12].

In addition to epithelial cell dysfunction, a type 2 inflammatory pattern involving expression of interleukins (IL) IL-4, -5, and -13 and increased concentrations of IgE, has been reported in the NPs of 85% of patients with CRSwNP in western countries [13]. Evidence of high levels of IL-6 expression has already been reported in NPs [14, 15]. IL-6 plays an important role in the development and progression of inflammatory responses, autoimmune diseases, and cancers. IL-6 can induce tissue damage, inflammation and cell proliferation [16–18]. To date, no study has precisely described the role of IL-6 in CRSwNP, and particularly its effect on mucociliary clearance, although one study does describe the effect of IL-6 on the regeneration of airway ciliated cells from basal stem cells [19]. More recently, high concentrations of IL-9 and IL-10 have been described in NPs but their influence on nasal airway epithelial cell dysfunction are unknown [16, 20]. Our hypothesis was that inflammatory cytokines in NPs, particularly IL-6, could be responsible for alteration of sinonasal epithelial cell functions (i.e. dysfunction of repair mechanisms and mucociliary clearance) thus creating favorable conditions for chronic inflammation and polyp growth.

We thus set out to investigate in vitro the relationship between nasal epithelial cell functions and ILs. We developed air-liquid interface (ALI) cultures of primary differentiated human nasal epithelial cells (HNEC) that can be used as an in vitro wound repair and ciliary beating evaluation model. Our results suggest new mechanisms of epithelial cell-IL relationships and may lead to the identification of novel therapeutic pathways that could improve treatment for patients with CRSwNP [8].

## Methods

In healthy conditions, after a mechanical wound, epithelium repair mechanisms involve cell migration, followed by a cell proliferation phase, epithelial junction and finally a differentiation phase of basal cells in ciliated cells [21]. The restoration of barrier integrity and mucociliary clearance after epithelial injury represent a key step in the defense capacity of the airway epithelium [11]. We aimed to evaluate these mechanisms of epithelial repair with and without IL modulation in cultures of HNEC from NPs.

### Primary Cultures of Human Nasal Epithelial Cells (HNEC)

NPs were obtained from 11 patients with CRSwNP undergoing ethmoidectomy. CRSwNP is a heterogeneous inflammatory disease with various underlying pathophysiologic mechanisms which correspond to different endotypes and clinical manifestations of the disease [22]. In this study, our samples were obtained from the most severe patients, i.e. those with medically uncontrolled CRSwNP and needing surgery. However, to ensure the homogeneity of the samples, all patients were required to stop oral corticosteroids treatment 1 month before surgery, and in all cases, surgery was decided after at least 3 months of well-conducted medical treatment with daily intranasal corticosteroids. All the patients had given informed consent and the study was approved by the local ethics committee (CPP IDF X 2016-01-01). HNECs were isolated from NPs as previously described [23]. Briefly, the NPs were immediately placed in DMEM/F-12 supplemented with antibiotics (100 U/ml penicillin, 100 mg/ml streptomycin, 2.5 g/ml amphotericin B, and 100 mg/ml gentamicin) and sent to the laboratory for processing. Enzymatic digestion [0.1% (wt/vol) pronase in culture medium] was performed for 16 h at 4 °C. The HNECs (1 × 10^6^ cells per well) were then plated in inserts (12-mm Transwell; Costar, MA) with 12-mm-diameter polycarbonate micro-pore membranes (pore size of 0.4 µm) coated with type IV collagen (Sigma), and incubated at 37 °C in 5% CO2. For the first 24 h, the cells were incubated with 1 ml of DMEM/F-12-antibiotics with 2% Ultroser G in the lower chamber and DMEM/F-12-antibiotics with 10% FCS in the insert. After 24 h, the culture medium (Stemcell, Pneumacult -ALI medium) in the insert was removed to place the cells at the ALI. The medium in the lower chamber was then changed every day. The epithelial nature of the cultured cells has been already confirmed by flow cytometric analysis of cytokeratin immunofluorescent labeling showing 95% and 99% of positive cells on Days 3 and 7, respectively [24]. The HNECs reach a stable differentiated state with the detection of ciliated, secretory, and basal cells during the third week of culture (Day 21) with the ALI culture medium (Stemcell, Pneumacult -ALI medium) [25]. The ALI model of culture is a polarized model (i.e., apical towards the air and basal towards the medium) where HNECs are exposed to air, as in the nose. This ALI model of differentiated HNECs therefore closely mimics in vivo conditions, except that they are not in their natural environment, apart from the potential inflammatory process occurring in the nasal cavity [24]. It constitutes an excellent tool to study cellular and molecular mechanisms involving airway epithelial cells exposed to various stimuli [26, 27]. This model is therefore highly suitable for the study of IL effects on epithelial airway repair and ciliary beating.

### In vitro wound repair model

The in vitro wound repair assay was carried out according to a model of mechanical injury adapted from a previously described method on fully differentiated cultures of HNECs [28]. We introduced a controlled linear wound of 12 mm length × 1.4 mm by scraping the HNECs with a pipette tip, followed by extensive washing to remove cellular debris. To determine the repair rate, time-lapse images were taken at regular intervals with an inverted microscope (Zeiss, Axiovert200M, France) equipped with an ×10 objective over a period of 48 h, depending on the time of wound closure (H18, H24, H42, H48 after wound healing). The wound areas were then quantified by image analysis software (*Image J.*). Wound closure was evaluated by the percentage of wound repair calculating the ratio between the wounded area at each time point and the initial wounded area. The reproducibility of the wound was evaluated by comparing the initial wound area of each tested condition.

### Effect of IL-6, IL-5, IL-9, IL-10 on wound closure

Closure in the presence of exogenous ILs was compared with closure without ILs (unstimulated control) in a context of serum-free and cytokine-free medium. Immediately after wounding, the HNECs were exposed to *DMEM*-*HAM*-*F12*-*Penicillin*–*Streptomycin*–*Fungizone*–*Gentamycin*–*free medium* with either IL-6 (1 ng/ml) or IL-5 (200 ng/ml) or IL-9 (20 ng/ml or IL-10 (100 ng/ml) or without IL. These concentrations were chosen according to previous studies that showed an IL effect on epithelial cells [16, 29–35].

The choice of ILs included in our study was made as follows. The TH2 cytokine IL-4 was not chosen as its role in nasal epithelial wound repair is already well described and it decreases wound closure in vitro [29]. IL-6 was selected because this cytokine has been shown to accelerate the wound closure rate in human biliary epithelial or corneal cell injury models and it could play a key role in dysregulation of repair mechanisms [32, 35]. IL-5 is one of the cytokines that characterizes the TH2 (eosinophilic) inflammatory pattern which increases recruitment and survival of eosinophils and its level of expression could be associated with severe recurrent forms of NPs [36, 37]. As a previous study showed no effect of IL-5 on epithelial repair, it was selected for this assay as a negative control hypothesis [29]. Inflammatory cell (i.e., Th2 cells, mast cells and eosinophils) secretion of IL-9 is also elevated in CRSwNP, particularly when associated with allergy and asthma [20, 38]. IL-9 participates in the expression of the IL-5 receptor and acts synergistically with IL-5 to reduce apoptosis of eosinophils in asthma [39, 40]. These features make this cytokine a potential candidate in the modulation of inflammatory response in patients with CRSwNP. However, its effects on epithelial cells are not well defined [20, 38]. IL-9 could induce changes in epithelial cell gene expression leading to goblet cell metaplasia and mucus overproduction [34, 38, 41]. We thus wanted to test its effect on epithelial repair. Finally, according to recent studies, IL-10, a potent anti-inflammatory cytokine, could also have a pivotal role in the pathogenesis of CRSwNP [16]. There is a need to investigate the role of IL-10 in the pathogenesis of NP and potentially to verify the hypothesis that IL-10 could counteract the dysregulation of repair mechanisms. IL-6 was chosen to investigate a dose–response effect on HNEC repair. For this, we added IL-6 to the cultures at increasing concentrations (1 ng/ml, 10 ng/ml, 100 ng/ml) 1 h after wounding for 48 h (i.e., until the repair assay). Each IL-6 concentration was tested in triplicate for each culture from the same patient. Results are expressed as the residual denuded area covered after each interval: [(initial wound area − residual wound area)/initial wound area] corresponding to the rate of repair (%).

### Effect of IL-6, IL-5, IL-9, IL-10 on differentiation process after wound closure

The effects of the ILs on differentiation in the HNEC cultures after wound closure (2, 7 and 12 days after wound closure) were characterized by immunolabeling. In this part of the study the culture wells were modulated from just after wound closure to 12 days after wound closure by each IL [(IL-6 (1 ng/ml), IL-5 (200 ng/ml), IL-9 (20 ng/ml) or IL-10 (100 ng/ml)], or not (unstimulated control wells) in order to only evaluate the effect of IL on differentiation process. After each IL-stimulation, we assessed cellular toxicity using both the TEER (Transepithelial electrical resistance) measurement and trypan blue staining for cellular viability 1 day before immunolabeling. The wells were fixed with 4% paraformaldehyde for 15 min at room temperature, washed three times with phosphate-buffered saline (PBS)++ (i.e., PBS supplemented with 0.4 with: 9 mM MgCl_2_ and 0. with: 9 mM CaCl_2_), incubated for 10 min with PBS++ NH_4_Cl 5 with: 0 mM and then permeabilized with 0.1% Triton X-100 for 10 min. After rinsing twice with PBS++, each well was separated in three areas for each immunolabeling marker before IL-staining in order to avoid mixed antibodies contaminations (by cutting each well in three areas). The cells were incubated either with mouse monoclonal anti-cytokeratin 14 (CellMarque 314M-14, 1/100) as a basal cell marker, mouse monoclonal anti-mucin-5AC (Abcam ab3649, 1/200) as a goblet cell marker, or mouse monoclonal anti-acetylated α-tubulin (Abcam ab24610, 1/500) as a ciliated cell marker, before being revealed by a secondary goat anti-mouse Alexa Fluor- 488 antibody (Molecular Probes 1/500). All the antibodies were incubated with 1% bovine serum albumin (Sigma-Aldrich, Darmstadt, Germany) in PBS++. The cells were finally washed three times with PBS++, mounted in ProLong^®^ Gold Antifade Reagent with 4′,6-diamidino-2-phenylindole (DAPI (Cell Signaling #8961), and imaged on a Zeiss LSM 700 scanning laser confocal microscope (Carl Zeiss MicroImaging GmbH). Two negative controls were performed either by omitting the primary antibody or by using non-immune mouse serum.

### Effect of IL-6 on ciliary beating

We went on to evaluate the IL-6 effect on ciliary function. Cilia differentiation was evaluated in cultures of HNEC either exposed or not to IL–6 when monolayers of basal cells were confluent after 3 or 4 days of culture. IL-6 was added in the culture medium at different concentrations (1 ng/ml, 10 ng/ml, and 100 ng/ml) during the differentiation phase of culture. On Day 21, culture brushing was performed and the cells were suspended in a survival medium (*DMEM*-*HAM*-*F12*-*Penicillin*–*Streptomycin*–*Fungizone*–*Gentamycin*) to immediately evaluate ciliary beating parameters: the ciliary beat frequency (CBF) (in Herz), the cilia length (in μm), the relative ciliary density (in %), the metachronal wavelength (in μm), and the ciliary beating efficiency index (in mPa). All experiments were performed in duplicate on the 11 independent cultures.

### Digital high-speed video-microscopy and evaluation of ciliary beating parameters

All in vitro ciliary function analyses were performed at controlled room temperature (20–25 °C) in the laboratory. We used an inverted microscope in brightfield conditions associated with a ×40 objective and a x100 objective. 20 μL of 4.5 μm polystyrene microbeads at a concentration of 0.125%w/v were added to 80 μL of survival medium containing beating ciliated cells in suspension and placed between a microscope slide and a cover slide. Cilia movements were recorded with a digital camera (PixeLINK A741, Ottawa Canada) at a rate of 358 frames per second. Each movie was composed of 1800 frames with a definition of 256 × 192 pixels, each individual pixel being (0.32 × 0.32) μm^2^ with a ∂40 objective and (0.13 × 0.13) μm^2^ with a ×100 objective. All areas containing intact undisrupted ciliated epithelial edges greater than 50 μm, beating in the plane of the camera were recorded. The parameters—CBF, cilia length, the relative ciliary density, the metachronal wavelength, and the ciliary beating efficiency—were recorded and analyzed in the ciliated edges of 10 different cell clusters with the ×40 objective for all except for the cilia length that was measured with the ×100 objective.

All the parameters were studied using analysis software developed by our team from the Matlab platform. The CBF (in Hz) was obtained with the temporal analysis of the grey level by Fast Fourier Transform. The relative ciliary density was assessed by comparing the average grey level of the background with the grey level of the area containing cilia. Since ciliated areas are darker than the background, the higher the percentage, the higher the relative ciliary density. The metachronal wavelength (in µm) was obtained by inferring the phase variation of the main beating frequency along the ciliated edge. This phase shift, which expresses the delay of one cilium in the cycle compared to the neighbouring cilia, creates a metachronal wave that participates in the movement of mucus. The length of the cilia (in µm) was measured on the most rectilinear cilia for which the base, apical extremity and entire path were visible. Finally, microbead velocity was used as a marker of the flow generated by the ciliary beating to evaluate the shear stress induced by cilia on the fluid [mean ciliary efficiency index (in mPa)]. All parameters and measurement details have been previously published by our research team [42, 43].

### Statistical analysis

Statistics were performed with a statistical software package (Statistica v7.1; Stat Soft, France). Data were expressed as mean ± standard deviation (SD). Comparisons of repair rate between IL-stimulated conditions and unstimulated controls were performed with Kruskal -Wallis (unpaired data and quantitative variables). After observing the decrease in wound-closure area with a range of IL6-concentrations, we tested linear regression using the least-square method of estimation, which was shown to be appropriate. Comparison of proliferation (number of nuclei), differentiation (number of positive cells for each immunolabeling marker) between the IL groups and controls were performed with Kruskal -Wallis (unpaired data and quantitative variables).

Comparisons of mean quantitative parameters of beating between IL6 groups (at different concentrations) and unstimulated controls were performed with Friedman ANOVA by Rank with Wilcoxon test as post hoc test (paired data and quantitative variables). A p value ≤ 0.05 was considered as significant.

## Results

In CRSwNP pathogenesis, the initial mechanism in the formation of polyps could be a rupture of both the epithelial continuity and the basement membrane. A defective epithelial barrier has already been found in patients with CRSwNP with decreased tissue resistance and decreased expression of tight junction proteins [10]. Extrinsic factors could serve as environmental inflammatory triggers (inhaled irritants or particles, pneumo-allergens, commensal and pathogenic bacteria) which induce epithelial damage (barrier dysfunction) and inflammatory mechanisms [2, 3]. However, restoring the integrity of the epithelial barrier after injury is also a key element in the defence capabilities of the respiratory epithelium. Dysfunction of repair of the epithelial barrier has been implicated in the pathogenesis of NPs [3, 11, 12]. However, the mechanisms leading to epithelial cell dysfunction remain poorly understood. Our hypothesis was that among the inflammatory cytokines involved in NPs, IL-6 could alter epithelial repair mechanisms.

### In vitro IL effect on wound closure

**Fig. 1:**
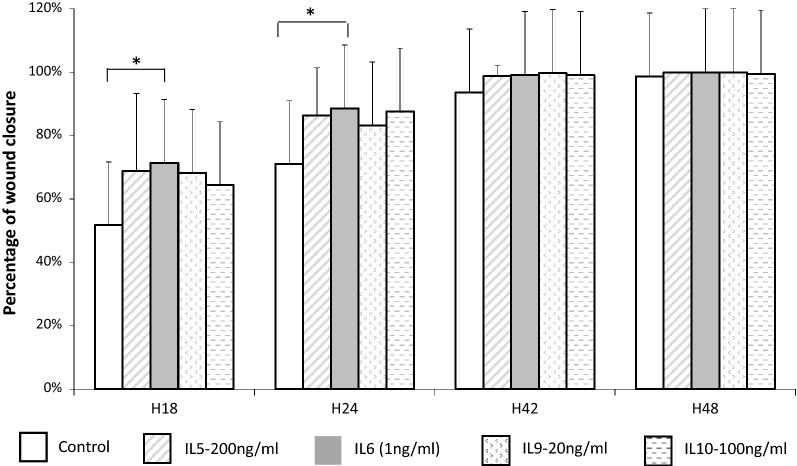
Study of the effect of ILs on wound closure. Comparison of the mean percentage of wound repair after wound healing of HNEC cultures (n = 11 patients) at each regular interval between control wells and each IL tested (IL-5, IL-6, IL-9, IL-10). *p value < *0.05*

We first evaluated the in vitro effect of the ILs on wound closure on 11 independent cultures corresponding to 102 wells which were wounded (IL-5:n = 15, IL-6:n = 17, IL-9 n = 20, IL-10 n = 14 and unstimulated controls n = 36) (Fig. 1). IL concentrations were determined according to previous in vitro studies: IL-6 (1 ng/ml), IL-5 (200 ng/ml), IL-9 (20 ng/ml) or IL-10 (100 ng/ml). There was no statistical difference between initial wound areas according to each tested condition (p = 0.11). After 18 h, only the IL-6 group had a higher repair rate than the unstimulated control group (71 ± 22% vs 52 ± 23%, p = 0.02). Similarly, only the IL-6 group had a higher repair rate than the unstimulated control group after 24 h (89 ± 13% vs 71 ± 20%, p = 0.03). No significant effect on wound repair rate was observed for the other ILs tested (Fig. 1).

Based on the epithelial wound time experiments, we identified the cytokine with the greatest discrepancy among the four individual IL exposure groups. Only IL-6 exposure resulted in faster wound closure rates than the controls. IL-6 was thus chosen to investigate a dose–response effect on HNEC repair.

**Fig. 2:**
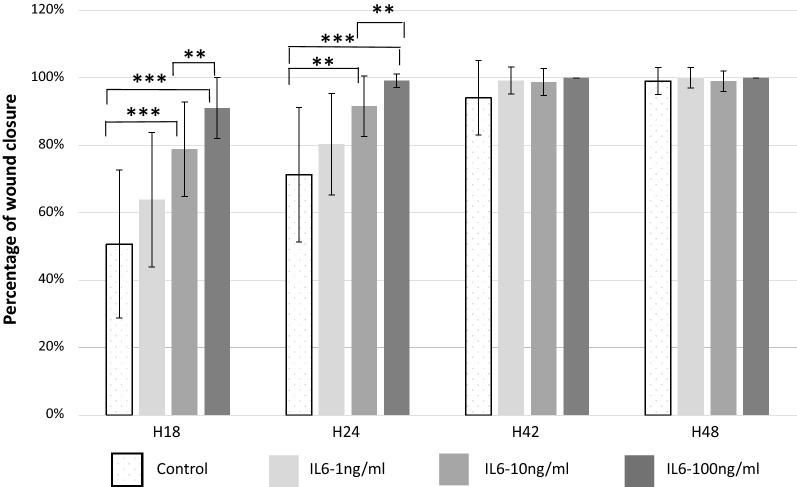
Study of IL6 dose–response effect on wound closure. Comparison of the mean percentage of wound repair after wound healing of HNEC cultures (n = 6 patients) at each regular interval between controls wells and each concentration of IL-6 tested: IL6-1, IL6-10; IL6-100 (1 ng/ml, 10 ng/ml, 100 ng/ml, respectively). *p value < *0.05;* **p < *0.005;* ***p < *0.0005*

The dose–response effect of IL-6 was evaluated on independent cultures (n = 6), corresponding to 166 wells (unstimulated control wells n = 48, IL6-1 ng/ml n = 29, IL6-10 ng/ml n = 22 IL6-100 ng/ml n = 19 wells). After 18 h, a significant increase in the mean wound repair rate was observed according to increasing concentrations of IL-6 compared to unstimulated control group (IL6-1: 75 ± 20%; IL6-10: 84 ± 14%; IL6-100: 91 ± 9%; vs 51 ± 21% in unstimulated control group, p < 0.0005) (Fig. 2). After 24 h, 79% of wounded wells with IL6-100 (n = 15/19) were fully repaired, vs 46% in the IL6-10 group (n = 10/22), 28% in the IL6-1 group (n = 8/29), and 15% in the unstimulated control group (n = 7/48).

### In vitro IL effect on differentiation after wound closure

After a mechanical wound, epithelium repair mechanisms involve cell migration, followed by cell proliferation, and finally by ciliated and goblet differentiation of basal cells. Thus, in the second part of the study we evaluated the in vitro IL effect on cell proliferation (i.e., nuclei quantification) in the wound area, and the effect on cell differentiation (i.e., specific immunolabeling) at 2, 7 and 12 days after wound closure. The effect of the ILs on wound closure was evaluated on 10 independent cultures corresponding to 118 wells (IL-5 n = 9, IL-6 n = 30, IL-9 n = 23, IL-10 n = 9 and control wells without IL n = 35).

#### Evaluation in non-wounded control areas

No difference was observed between IL-stimulated cultures and unstimulated control wells according to the number of nuclei, the proportion of ciliated cells, or goblet or basal cells whatever the duration of stimulation. We then compared the time-effect within each IL-group: in the IL-6, IL-9 and IL-10 stimulated groups, there was no significant difference between the three time points according to the number of nuclei, the proportion of ciliated cells, or goblet or basal cells. In the IL-5-stimulated group, we found a significant increase in the mean number of nuclei between Day 2 and Day 12 (481 ± 59 vs 390 ± 60, p = 0.03) (data not shown).

#### Evaluation in wounded areas

In the control wells without IL, a typical response to epithelial aggression was observed with a proliferation and differentiation process between Day 2 and Day 12. When comparing Day 2 to Day 12, we found a significant increase in the number of nuclei (270 ± 81 vs 327 ± 93, p = 0.01), in the proportion of ciliated cells (2 ± 2% vs 20 ± 3%, p < 0.00001), in the proportion of goblet cells (2 ± 2% vs 11 ± 3%, p < 0.00001) and a decrease in the number of basal cells (26 ± 5% vs 10 ± 3%, p < 0.00001) (data not shown).

**Fig. 3:**
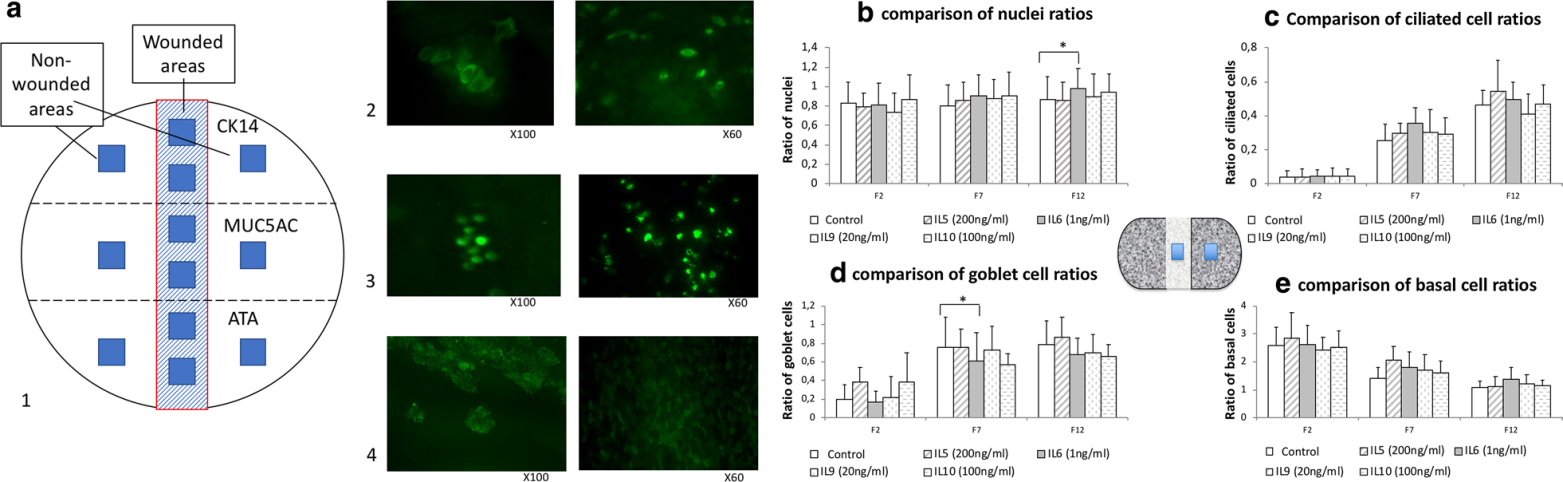
Study of the effect of ILs on the differentiation process after wound closure. **a** Diagram of well cutting and immunolabeling made in the three different thirds of wells (CK14 in the upper third, MUC5AC in the intermediate third and ATA in the lower third) and method of analysis: Effects of ILs on differentiation in HNEC cultures after wound closure (2 (F2), 7 (F7) and 12 (F12) days after wound closure) was characterized by immunolabeling: each well was separated in three areas for each immunolabeling marker before IL-staining in order to avoid mixed antibodies contaminations (by cutting each well in three areas): either with mouse monoclonal anti-cytokeratin 14 (CellMarque 314 M-14, 1/100) (picture **a**-1) as a basal cell marker, mouse monoclonal anti-mucin-5AC (Abcam ab3649, 1/200) (picture **a**-2) as a goblet cell marker, or mouse monoclonal anti-acetylated α-tubulin (Abcam ab24610, 1/500) (picture **a**-3) as a ciliated cell marker, before being revealed by a secondary goat anti-mouse Alexa Fluor- 488 antibody (Molecular Probes 1/500). Nuclei were stained by DAPI. In order to evaluate the “wound” effect and overcome the IL effect on healthy epithelium, we measured the ratio of the number of nuclei stained by DAPI or the percentage of cells positive for each immunolabeling (ATA, MUC5AC, and CK14) in two wounded areas on the number of nuclei for the DAPI in the corresponding two non-wounded control areas of the same well. **b**–**e** Comparison of ratios (percentage of positive cells in wounded area/Percentage of positive cells in non-wounded control areas) after 2, 7 and 12 days after wound closure between culture wells modulated by each IL (IL-5, IL-6, IL-9, IL-10) and not (controls wells). **b** Comparison of nuclei ratios (wounded areas/non-wounded areas) between culture wells with IL and without *p < 0.05. **c** Comparison of ciliated cell ratios (wounded areas/non-wounded areas) between culture wells with IL and without *p < 0.05. **d** Comparison of goblet cell ratios (wounded areas/non-wounded areas) between culture wells with IL and without *p < 0.05. **e** Comparison of basal cell ratios (wounded areas/non-wounded areas) between culture wells with IL and without *p < 0.05

According to the initial hypothesis of an epithelial repair defect in NP formation, we specifically evaluated the effect of ILs on the proliferation and differentiation at the level of the epithelial wound-closed area between each IL-stimulated group and the control group without IL stimulation. To rule out the IL-proliferation effect on healthy tissue, we calculated the ratio of number of nuclei between the wound area at two sites in the same well and the healthy non-wounded control area at two sites (Fig. 3a). We then calculated with the same method of analysis (described in Fig. 3a) the ratio of ciliated cells, goblet cells and basal cells at Day 2 and Day 12.

A similar differentiation process was observed in culture wells modulated by each IL between Day 2 and Day 12 (increase in nuclei, ciliated cells, goblet cells and decrease in basal cells) (Fig. 3). Only IL-6 stimulation affected the number of nuclei: there was a higher ratio of nuclei on Day 12 after wound closing (p < 0.05) compared to the wounded control wells without IL. No IL effect was observed on the ratio of ciliated cells, goblet cells or basal cells compared to the wounded control wells without IL.

### Evaluation of ciliary beating function after in vitro IL-6 stimulation

After restoration of barrier integrity after epithelial wounding, mucociliary clearance represents a key step in the defense capacity of the airway epithelium and could be altered in CRSwNP. Thus, we evaluated the ciliary function after in vitro IL-6 stimulation. A dose–response effect of IL-6 on ciliary beating was evaluated on 11 independent cultures stimulated or not by IL-6 for 21 days of culture corresponding to 79 wells (unstimulated control wells n = 21, IL6-1 ng/ml n = 18, IL6-10 ng/ml n = 19, IL6-100 ng/ml n = 21 wells).

**Fig. 4:**
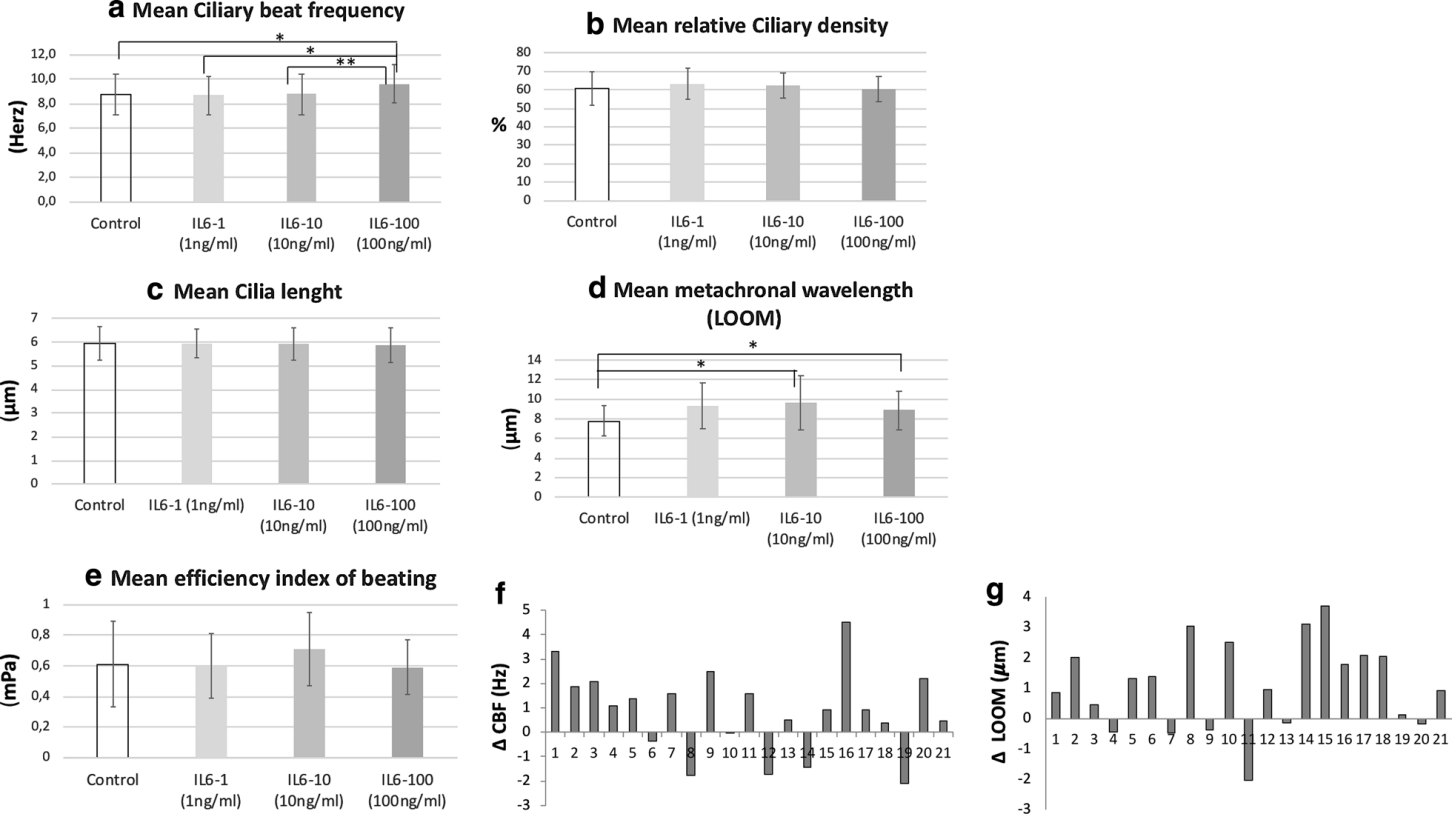
Study of IL-6 effect on ciliary beating. Comparison of ciliary beating parameters between IL-6 groups at increased concentrations (IL6-1, IL6-10, IL6-100) (1 ng/ml, 10 ng/ml, 100 ng/ml respectively)) and control group after 21 days of stimulation by IL-6 in culture medium of HNEC (n = 21 controls wells and n = 21 wells with IL6-100 and n = 21 wells with IL6-10 and and n = 21 wells with IL6-1) (paired analysis): comparison of mean ciliary beat frequency (CBF) (in Herz) (**a**), mean relative ciliary density (**b**), mean cilia length (**c**), mean metachronal wavelength (LOOM) (in μm) (**d**), mean efficiency index of beating (in mPa) (**e**). **f**, **g** Focus of significant results of paired analysis: variation of ciliary beat frequency (CBF) between each well with IL-6 stimulation at 100 ng/ml and its respective control well (n = 21) (**f**) and focus on variation of metachronal wavelength (LOOM) between each well with IL-6 stimulation at 100 ng/ml and its respective control well (n = 21) (**g**)

The mean CBF was 8.8 Hz in the control wells and did not significantly differ from that observed in the IL-6 groups (IL6-1: 8.7 Hz, IL6-10: 8.8 Hz and IL6-100: 9.6 Hz) (Fig. 4a). However, for each culture, the paired analysis of each well with IL-6 at different concentrations with its unstimulated control well showed a higher CBF in IL6-100 compared to the paired unstimulated control well (p = 0.042) and the paired IL6-1 and IL6-10 wells (p = 0.039 and p = 0.004, respectively) (Fig. 4a, f).

The mean metachronal wavelength was 7.8 μm in unstimulated control wells, 9.3 μm for IL6-1, 9.7 μm for IL6-10 and 8.9 μm for IL6-100 (Fig. 4d). In the same way, the paired analysis showed a significantly higher metachronal wavelength in the IL6-10 and -100 groups compared to the paired unstimulated control wells (p = 0.007 and p = 0.01, respectively) (Fig. 4g).

The paired analysis for each of other parameters showed no significant difference in cilia length, relative ciliary density and ciliary efficiency index between the unstimulated control group and those exposed to each IL-6 concentration for 21 days (Fig. 4b, c, e).

## Discussion

Novel therapies to improve disease control are needed to spare patients from systemic corticosteroids and repeated sinus surgery. Monoclonal antibodies (mAbs) directed against IgE (omalizumab) or cytokines such as IL-5 (mepolizumab and reslizumab), and IL-4/IL-13 (dupilumab) have been tested as innovative therapeutic approaches of CRSwNP. Some mAbs have recently been proved in phase 3 trials as efficient therapy on nasal polyp size and reduction of severity of symptoms [44]. Improving the understanding of different endotypes should provide insight for determining appropriate current and new therapies [45–48]. Kim et al. concluded that endotypes depend on the epithelial barrier function, epithelial cytokines, and T cell subsets [46]. Consequently, in this study we chose to unravel the specific role of IL-6 in nasal polyposis as a potential target for new monoclonal therapies in CRSwNP. Indeed, the effect of IL-4/IL-13 and IL-5 on nasal epithelial repair was already showed [29] but to date, no study has precisely described the role of IL-6, IL-9 and IL-10 (new ILs described in nasal polyposis) on human nasal epithelial cells in an in vitro wound repair model.

Among the cytokines involved in the pathophysiology of CRSwNP—such as IL-5, IL-9 and IL-6 or the regulatory cytokine, IL-10—only IL-6 was found to have an effect on wound healing by significantly accelerating the wound closure rate. After wound closure, although none of the interleukins (IL-5, IL-6, IL-9, IL-10) altered the differentiation process, the presence of IL-6 induced an increase in the ratio of nuclei (wounded area/non-wounded area) at late-stage repair (Day 12). IL-6 was also found to affect ciliary functions by increasing CBF and metachronal wavelength.

The mechanisms underlying the pathogenesis of CRSwNP remain poorly defined [37]. Various research groups have focused on exploring the roles of sinonasal epithelial cells, of the host immune system, and of pathogens (i.e., bacteria) [37]. In a new concept, whatever the initial factor of aggression (exposure to inhaled pathogens, antigens or particulates), injury of the nasal airway epithelial barrier triggers a chronic nasal inflammatory process leading to the development of CRSwNP [49]. After that, cytokines and the dysregulation of the host immune response may result in an abnormal epithelial remodeling process and to NP growth [14]. Novel therapies are required to improve disease management and thus avoid treatment by systemic corticosteroids and repeated sinus surgery [49]. According to the epithelial hypothesis it is necessary to understand the role of inflammatory factors on epithelial cells to identify the key mediators of the disease and consequently define new targets for emerging biological therapies.

In the present study, we confirmed our initial hypothesis, i.e. that the lack of IL-5 does not affect epithelial repair mechanisms as suggested in the literature [29]. On the other hand, IL-5 did increase the number of nuclei in non-wounded “healthy” epithelium due either to an increased epithelial cell proliferation or to an inhibition of epithelial cell apoptosis. It has recently been shown in Alzheimer’s disease that IL-5 blocks the apoptosis of neural cells [31]. We also found that IL-9 did not affect goblet cell differentiation as we might have expected in view of its known role in metaplasia goblet cell induction. On the other hand, we confirmed our hypothesis that IL-6 has an effect on airway epithelial repair mechanisms: IL-6 induced an increased wound closure rate similar to the effect observed in human biliary epithelial or corneal cell injury models [32, 35]. This study also showed quantitative up-regulation of cell proliferation with IL-6 in the case of prior epithelial damage compared with other IL-stimulated conditions.

While some authors [15, 37] suggest that IL-6 plays an important role in chronic inflammation in CRSwNP, its mechanism of action remains poorly understood. The role of IL-6 in NP pathophysiology is classically described as an adaptive immune response by inducing B cell proliferation and activation and neutrophil recruitment [17]. However, we found that IL-6 could also be involved in other roles such as up-regulation of epithelial cell proliferation and partial ciliary beating control.

IL-6 is produced by various types of cell such as T-cells, B-cells, monocytes, fibroblasts, keratinocytes, endothelial cells, mesangial cells, adipocytes and some tumor cells [18]. In CRSwNP, the initial extrinsic factor of aggression such as fungi, bacteria and lipopolysaccharides (the main outer surface membrane component of Gram-negative bacteria) could explain the increase in IL-6 expression in NPs [50]. Up-regulation of IL-6 may be also explained by increased fibroblast activity dependent on an ongoing chronic local inflammation possibly initiated by an infection [14].

At a molecular level, IL-6 is known to increase vascular permeability and angiogenesis during the acute response phase following wound healing by inducing VEGF production [18]. It can induce the T-helper cell 17 (Th17) immune response by binding to a specific receptor, IL-6R, which triggers a cascade of signaling events that result in STAT3 activation and gene transcription culminating in Th17 differentiation and IL-17 production [51]. STAT3 also regulates the process of cell migration and is therefore important for wound healing. The STAT3-effect is in agreement with the in vitro effect of IL-6 in our study. Using IL-6-deficient mice model, a study reported that the IL-6 system was pivotally involved in epidermal permeability barrier repair via STAT3 [52]. In line with our results in a chronic inflammation model, IL6 has also been reported [33] to increase epithelial tumoral cell proliferation, migration and invasion in an ovarian cancer model. Moreover, they also showed that the pro-tumoral effects can be reversed by inhibiting IL-6 both in vivo and in vitro [53]. Along the same lines of the IL-6 effect in the cancer model, IL-6 could induce up-regulation of proliferation in CRSwNP in the case of “wounded” epithelium. IL-6 exerts its biological activities through two molecules: IL-6R—also known as IL-6Rα—and gp130—also known as IL-6Rβ [18]. The intra-cytoplasmic domain of gp130 contains motifs that can activate both the JAK/STAT and the ERK1/2 (extracellular-signal-regulated kinase) signaling pathways. Activation of the JAK/STAT pathway induces, for instance, activation of NFκB which can block cell apoptosis, and which could explain the increase in epithelial cells in our HNEC model. Activation of the ERK1/2 pathway can induce the production of metalloproteases and tumor cell proliferation [18]. We have already described in our wound repair model that metalloproteases such as MMP-2 can induce acceleration of wound repair in vitro [28]. Thus, the IL-6 effect observed in our study could be explained by the up-regulation of metalloproteases.

Interestingly, IL-6 can also associate with the naturally occurring soluble IL-6R (sIL-6R). The IL-6/sIL-6R complex can thereby activate target cells which do not express the membrane bound IL-6R. Such cells would not be able to respond to IL-6. This process has been called ‘‘trans-signaling’’ [54]. Since all human cells express gp130 on the cell surface, the IL-6/sIL-6R complex can activate nearly all body cells. In other chronic inflammatory diseases such as inflammatory bowel disease, peritonitis, rheumatoid arthritis, asthma as well as in colon cancer, IL-6 trans-signaling is critically involved in the maintenance of the disease state [17]. In an inflammatory colon cancer model, stimulation by the IL-6/sIL-6R complex induced intra-tumoral growth of epithelial cells [17]. Moreover, in all these animal models, the progression of the disease could be interrupted by specifically interfering with IL-6 trans-signaling using recombinant-soluble gp130Fc protein. Therefore, the in vitro IL-6 effect observed in our study could be explained by activation of its different cell signaling pathways and its working via the trans-signaling function. In conclusion, IL-6 trans-signaling could induce chronic proliferation of epithelial cells after wound healing of the epithelial barrier and growth of NPs.

Finally, mucociliary dysfunction could be a prominent pathophysiological feature of CRSwNP [8, 55, 56]; however, the precise mechanisms underlying mucociliary dysfunction are still unclear. A previous study demonstrated in the same in vitro primary model of cultures that IFN-γ and IL-13 both significantly reduced ciliated cell differentiation and CBF in CRSwNP patients [57]. Our study showed an opposite effect of IL-6 which increased the CBF and the metachronal wave but without modifying the ciliary beating efficiency. Ciliary function is a dynamic process and can be modified by exogen stimuli in physiological conditions [58]. These variations observed under IL-6 modulation could correspond to an attempt to adapt ciliary function by increasing coordination and velocity of beating but without success. There is only one study to our knowledge that has studied the IL-6 effect on CBF and which showed an opposite effect with a decreased beat frequency [59]. It was an isolated study conducted on fallopian tube mucosa explants, thus limiting the comparison with the ciliary beating of an airway epithelium in contact with air. Our study therefore presents the first in vitro results of an IL-6 effect on the ciliary beating in human nasal epithelial ciliated cells. Mucociliary clearance is one of the major lines of defense of the respiratory system. Overall, the epithelial barrier dysfunction could be involved in physiopathology of nasal polyps [8, 10, 60]. Further studies are needed to evaluate the IL-6 effect on mucosal barrier (especially on expression of tight junctions and monolayer permeability).

## Conclusion

In summary, CRSwNP can be regarded as a multifactorial disease resulting from abnormal epithelial repair that could be IL modulated. Here, primary HNEC cultures revealed a relationship between IL-6 and epithelial cells, suggesting that IL-6 could be involved in the growth of nasal polyps. Epithelial wound closure is significantly faster and CBF is significantly increased under IL-6 stimulation, which could induce an exaggerated epithelial response to external aggression and, in turn, promote polyp formation. This information could be useful in developing new research and therapeutic approaches regarding epithelium dysfunction in CRSwNP.

## Data Availability

All data generated or analysed during this study are included in this published article [and its additional files].

## Abbreviations

ALI: Air–liquid interface
CBF: Ciliary beating frequency
CRSwNP: Chronic rhinosinusitis with nasal polyps
HNEC: Human nasal epithelial cells
IL: Interleukin
NPs: Nasal polyps
